# Targeting PVT1 Exon 9 Re-Expresses Claudin 4 Protein and Inhibits Migration by Claudin—Low Triple Negative Breast Cancer Cells

**DOI:** 10.3390/cancers13051046

**Published:** 2021-03-02

**Authors:** Fayola Levine, Olorunseun O. Ogunwobi

**Affiliations:** 1 Department of Biological Sciences, Hunter College of The City University of New York, New York, NY 10065, USA; FAYOLA.LEVINE20@myhunter.cuny.edu; 2 The Graduate Center Departments of Biology and Biochemistry, The City University of New York, New York, NY 10016, USA; 3 Joan and Sanford I. Weill Department of Medicine, Weill Cornell Medicine, Cornell University, New York, NY 10021, USA

**Keywords:** PVT1, triple negative breast cancer, claudin-low

## Abstract

**Simple Summary:**

Triple negative breast cancer accounts for 10–15% of all breast cancers. Specific molecular characteristics have led to the identification of six subtypes of triple negative breast cancer, with one in particular being claudin-low. PVT1, a non-protein coding gene, has been demonstrated to play an oncogenic role in various cancers. Specifically, PVT1 exon 9 has been shown to have oncogenic capability. In this study, we aimed to assess the role of PVT1 exon 9 in triple negative breast cancer cells. We have observed that siRNA targeting of PVT1 exon 9 in claudin-low triple negative breast cancer cells resulted in re-expression of claudin 4 protein, and inhibition of migration. These findings indicate that PVT1 exon 9 regulates claudin 4 expression and migration in triple negative breast cancer cells.

**Abstract:**

PVT1 is a long non-coding RNA transcribed from a gene located at the 8q24 chromosomal region that has been implicated in multiple cancers including breast cancer (BC). Amplification of the 8q24 chromosomal region is a common event in BC and is associated with poor clinical outcomes. Claudin–low (CL) triple negative breast cancer (TNBC) is a subtype of BC with a particularly dismal outcome. We assessed PVT1 exon 9 expression in the T47D estrogen receptor positive BC cell line, and in the MDA MB 468 and MDA MB 231 TNBC cell lines, followed by the assessment of the expression of claudins 1, 3, 4 and 7, in MDA MB 468 and MDA MB 231 (TNBC) cells. We found that MDA MB 231 TNBC cells significantly express less claudin 1, 3, 4, and 7 than MDA MB 468 TNBC cells. PVT1 exon 9 is significantly upregulated in MDA MB 231 CL TNBC cells, and significantly downregulated in MDA MB 468 claudin high (CH) TNBC cells, in comparison to T47D estrogen receptor positive BC cells. We then analyzed the functional consequences of siRNA targeting of PVT1 exon 9 expression in the MDA MB 231 CL TNBC cells. Notably, siRNA targeting of PVT1 exon 9 expression in the MDA MB 231 CL TNBC cells led to a significant reduction in migration and the re-expression of claudin 4. Taken together, our data indicate that PVT1 exon 9 regulates claudin 4 expression and migration in CL TNBC cells, and may have clinical implications in CL TNBC.

## 1. Introduction

Breast cancer is extraordinarily common worldwide. In 2018, breast cancer was the fifth leading cause of cancer related deaths globally [1]. In the United States, breast cancer is the second leading cause of cancer mortality in women [2,3] with a 13% lifetime risk of diagnosis and a 2.6% risk of death. An estimated 276,480 new cases of invasive breast cancer will be diagnosed in U.S women, of which close to 42,170 women will die from the disease in 2020 alone [2]. Breast cancer is classified according to the expression of three specific molecular markers; estrogen receptor (ER), progesterone receptor (PR) and human epithelial growth factor receptor 2 (EGFR2/HER2) [4]. Loss of these three receptors characterizes an intrinsic subtype of breast cancer called triple negative breast cancer (TNBC). TNBC accounts for 10–15% of all breast cancer cases and is typically more aggressive with poor clinical outcomes [5,6,7]. Based on molecular characteristics, TNBC can be further subdivided into additional subtypes. Based on gene expression profile study, six TNBC subtypes have been identified, each of which differs in histopathological features and their response to chemotherapy. These subtypes include basal-like 1 (BL1), basal-like 2 (BL2), immunomodulatory (IM), mesenchymal (M), mesenchymal stem-like/claudin low (MSL/CL), and luminal androgen receptor (LAR) [8]. Claudin–low triple negative breast cancer (CL TNBC) makes up 7–14% of all invasive breast cancers. Moreover, CL TNBC is associated with poor prognosis, and some studies report that they exhibit chemo-resistance [9,10,11]. 

**Figure 1:**
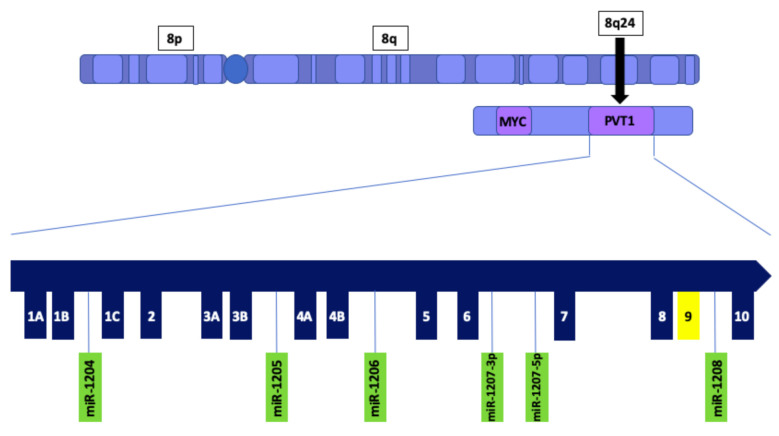
Schematic illustration of PVT1 showing exons and microRNAs. PVT1 is located downstream of the MYC gene on chromosome 8q24. PVT1 exon 9 is highlighted in yellow.

Plasmacytoma variant translocation 1 (PVT1) is a long noncoding RNA (lncRNA) that is transcribed from a gene located at the 8q24 chromosomal region and has been demonstrated to play an oncogenic role in multiple cancers including breast cancer [12]. The PVT1 gene is located approximately 53 kb downstream of the oncogene MYC [13], and contains several exons, including exons 1A, 1B, 1C 2, 3A, 3B, 4A, 4B, 5, 6, 7, 8, 9 and 10 [14] (Figure 1). Additionally, PVT1 encodes multiple alternatively spliced lncRNAs as well as six annotated microRNAs [15]. Alternative splicing is a tissue and cell specific mechanism, in which a diverse amount of mRNA isoforms is generated. Aberrant alternative splicing of pre-mRNAs is one of the characteristics of cancer [16]. Previously, we have reported that PVT1 exon 9 is overexpressed in prostate cancer cell lines, especially in aggressively tumorigenic prostate cancer cell lines derived from men of African Ancestry [17] Furthermore, we have observed that PVT1 exon 9 is significantly overexpressed in prostate cancer tissue relative to both normal prostate tissue and benign prostatic hyperplasia [18]. Also, we have demonstrated that PVT1 exon 9 induces malignant transformation and resistance to androgen deprivation therapy in prostate epithelial cells [19]. Although studies have shown that PVT1 splice variants are also overexpressed in breast cancer [20], and play a role in cancer progression [21,22], the underlying mechanisms by which these transcripts promote tumorigenicity is yet to be elucidated. PVT1 amplification is associated with many clinicopathological characteristics in breast cancer, including regulation of apoptosis [12], EMT [23] and metastasis [24]. Moreover, there is evidence that PVT1-derived transcripts also promote breast tumorigenesis [25,26,27]. Furthermore, PVT1 promotes breast tumorigenicity by modulating transcription factors that have been demonstrated to have oncogenic roles in cancer [28,29]. Further studies are required to elucidate PVT1’s role in TNBC, and other cancers.

Epithelial and endothelial cell-cell adhesion are mediated through multifunctional complexes known as tight junctions (TJ). The involvement of TJs in cancer biology is associated with dysfunctional signal transduction pathways that regulate cell-cell interactions [30]. Many studies have demonstrated that dysregulation of TJ proteins disrupts normal physiological function, which could lead to pathological consequences such as cancer [31,32,33,34,35]. Claudins (CLDNs) are a family of TJ proteins that consists of 27 members [36]. These tetraspan proteins contain an amino and carboxyl-terminal cytoplasmic domain, as well as two extracellular loops which are critical for maintaining TJ function [37]. CLDNs are tissue- or cell-specific and most tissues or cells express CLDNs in various combinations, or a single CLDN [38]. CLDN functions primarily involve maintaining cellular polarity, signaling [39], maintaining paracellular permeability [40,41] or paracellular channel [36]. Various studies demonstrate compelling evidence of CLDNs and their role in tumorigenicity. Both loss of function, and gain of function, of CLDNs in multiple cancers are well documented [42,43,44,45,46,47,48,49]. Claudin 4 (CLDN4) has been reported to be involved in various biological processes [50,51,52]. Patients with CLDN4 overexpression develop various clinicopathological characteristics including high tumor grade, poor prognosis and shorter disease-free survival. Additionally, it was reported that there is an association between ER status and CLDN4 expression in which ER- tumors significantly overexpressed CLDN4 [53,54]. Studies have shown that CLDN4 can be a useful prognostic marker in breast cancer [55,56]. Basal-like carcinomas, compared to tumor groups of grades 1–3, overexpressed CLDN4, while tumors of grades 1 and 2, displayed decreased, or absent, expression of CLDN4 [57]. While enhanced expression of CLDN4 in luminal breast cancers was linked to poor clinical outcomes, contrastingly, overexpression of CLDN4 in TNBC was associated with favorable outcomes in which tumors that overexpressed CLDN4 displayed a less aggressive phenotype [53]. Consequently, further research into molecular mechanisms regulating CLDN4 expression in triple negative breast cancer is warranted.

In this study we were interested in assessing the regulatory role of PVT1 exon 9 in CL TNBC cell line MDA MB 231. This has not been previously investigated. Altogether, our study revealed for the first time that targeting PVT1 in MDA MB 231 CL TNBC cells, specifically PVT1 exon 9, inhibits migration and induces re-expression of CLDN 4 in these cells.

## 2. Results

### 2.1. Claudins 1, 3, 4 and 7 Are Downregulated in Claudin Low Triple Negative Breast Cancer (TNBC) Cells

**Figure 2:**
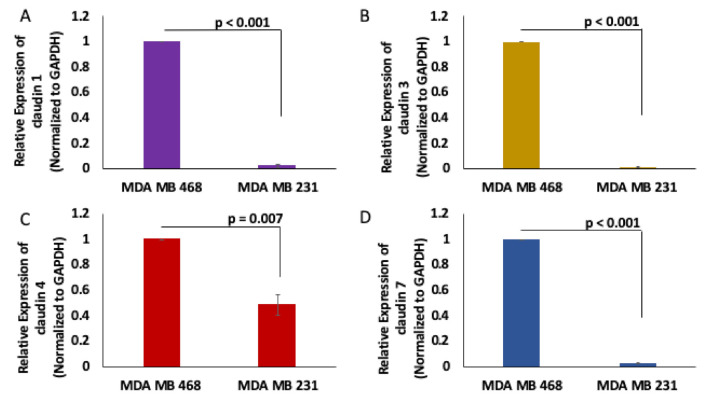
Claudins 1, 3, 4 and 7 expression in MDA MB 468 and MDA MB 231 cells. Claudin 1 (**A**), claudin 3 (**B**), claudin 4 (**C**) and claudin 7 (**D**) expression were assessed using real-time quantitative polymerase chain reaction in MDA MB 231 and MDA MB 468 cell lines; *N* = 4.

To determine the expression profile of claudins in TNBC, we assessed the expression of claudins 1, 3, 4 and 7 in the MDA MB 231 claudin–low TNBC cell line and the MDA MB 468 claudin–high TNBC cell line. We observed that claudins 1, 3, 4 and 7 are significantly downregulated in the MDA MB 231 CL TNBC cell line (Figure 2).

### 2.2. PVT1 Exon 9 Is Upregulated in Caludin- Low TNBC

**Figure 3:**
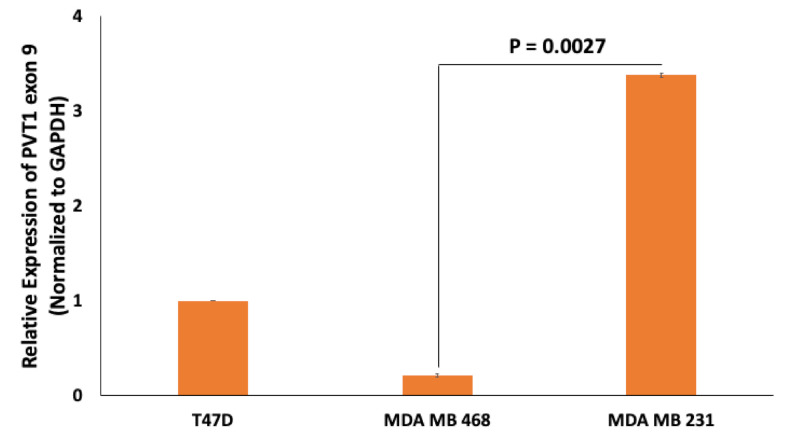
Comparison of PVT1 exon 9 expression in the T47D estrogen receptor positive BC cell line, MDA MB 468 CH TNBC cell line and the MDA MB 231 cell CL TNBC cell line. PVT1 exon 9 expression was assessed using RT-qPCR in the T47D estrogen receptor positive BC cell line, MDA MB 231 CL TNBC cell line and in the MDA MB 468 CH TNBC cell line. Expression was normalized against GAPDH. Data presented are from experiments performed in quadruplicates 6 separate times.

PVT1 exon 9 is overexpressed in prostate cancer [17] and promotes tumorigenicity by increasing proliferation and migration [19]. To assess the expression of PVT1 exon 9 in TNBC cell lines, we performed total RNA extraction, cDNA synthesis and real-time quantitative polymerase chain reaction (RT-qPCR). We observed that PVT1 exon 9 is significantly overexpressed in the MDA MB 231 CL TNBC cell line when compared to the MDA MB 468 CH TNBC cell line (Figure 3 and Figure S5). 

### 2.3. PVT1 Exon 9 Regulates Migration in Caludin- Low TNBC Cells

**Figure 4:**
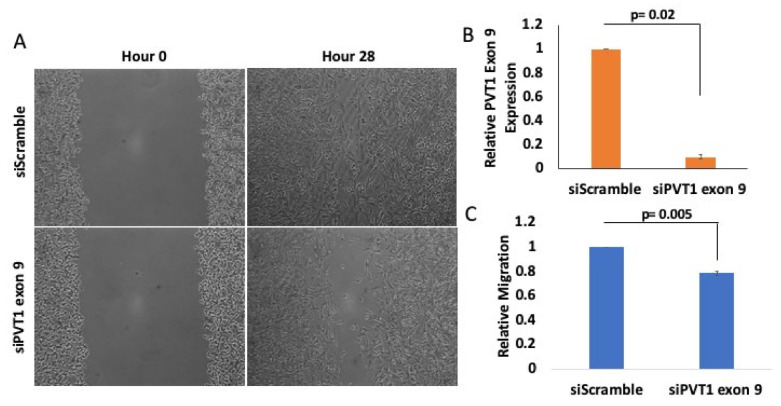
PVT1 regulates migration of MDA MB 231 CL TNBC cells. (**A**) Wound healing migration assays were performed with the MDA MB 231 CL TNBC cell line. MDA MB 231 cells were transfected once confluent. After 24 h, wounds were made and monitored between 0 h and 28 h. Images were taken at 10x magnification using Motic AE30 imaging software. (**B**) Knockdown of PVT1 exon 9 expression in the MDA MB 231 CL TNBC cell line at hour 0. Transfection of SiRNAs that specifically target PVT1 exon 9 was performed. Relative expression of PVT1 exon 9 in MDA MB 231 cells was assessed, based on data from 2 independent experiments. (**C**) Quantification of differences in migration, after 28 h, based on data from 3 independent experiments.

To determine if PVT1 exon 9 plays a role in the migration of MDA MB 231 CL TNBC cells, we performed wound healing migration assay. Short interfering RNA (SiRNA)-mediated knockdown of PVT1 exon 9 in MDA MB 231 CL TNBC cells significantly decreased migration, when compared to MDA MB 231 CL TNBC cells transfected with control scramble non-targeting SiRNA. Successful knockdown of PVT1 exon 9 in the MDA MB 231 CL TNBC cell line was verified by RT-qPCR. Assessment of the migratory capabilities of cells is based on the rate by which the wound closes. Pictures were taken at 0, 4, 24 and 28 h (Figure S4). We observed that MDA MB 231 CL TNBC cells in which PVT1 exon 9 was knocked down were significantly less migratory than MDA MB 231 CL TNBC control cells in which PVT1 exon 9 was not knocked down (Figure 4). This indicates that PVT1 is involved in regulating the migration of the MDA MB 231 CL TNBC cell line.

### 2.4. Targeting PVT1 Exon 9 Induces Re-Expression of Claudin 4 Protein in the Caludin- Low MDA MB 231 TNBC Cell Line

**Figure 5:**
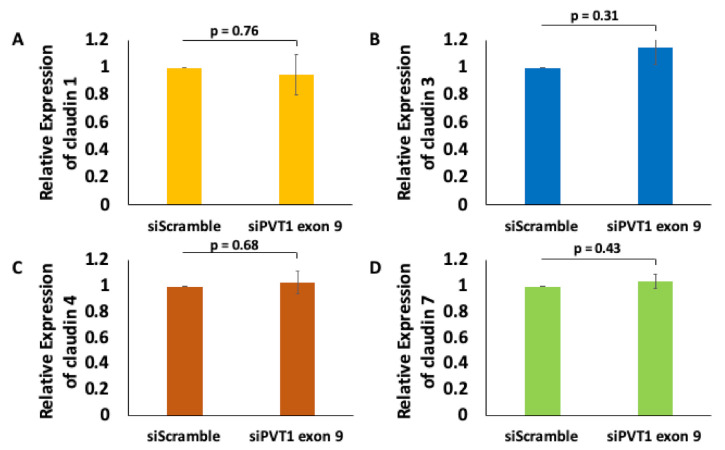
PVT1 exon 9 does not regulate mRNA expression of claudins 1, 3, 4, and 7 in MDA MB 231 cells. Claudin 1 (**A**), claudin 3 (**B**), claudin 4 (**C**) and claudin 7 (**D**) mRNA expression were assessed following knock down of PVT1 exon 9 in MDA MB 231 cell line. Data presented were normalized against GAPDH, and were obtained from three independent experiments.

**Figure 6:**
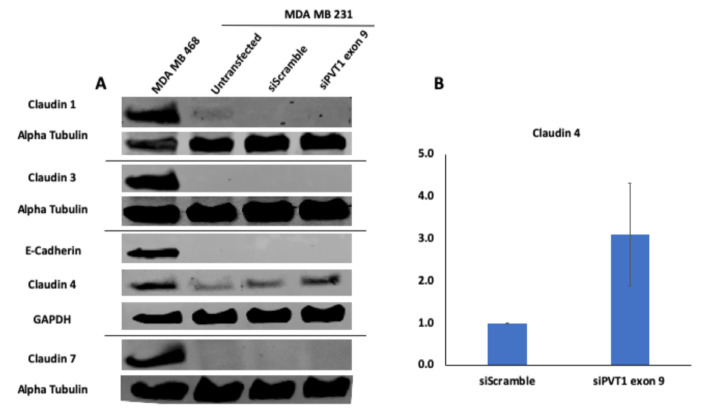
SiRNA targeting of PVT1 exon 9 induces claudin 4 protein re-expression in MDA MB 231 CL TNBC cells. (**A**) MDA MB 231 CL TNBC cells were transfected with PVT1 exon 9 specific siRNAs (siPVT1 exon 9) for 24 h. Western blotting was performed using specific antibodies against claudin 1, claudin 3, claudin 4, claudin 7 and E-Cadherin. SiRNA targeting of PVT1 exon 9 induced claudin 4 protein re-expression in MDA MB 231 CL TNBC cells in comparison to MDA MB 231 CL TNBC cells transfected with only control scramble non-targeting siRNA (siScramble). (**B**) Quantification of relative claudin 4 protein expression normalized to GAPDH protein expression; *N* = 2.

Given that the claudin–low MDA MB 231 TNBC cell line expresses significantly more PVT1 exon 9 than the claudin-high MDA MB 231 TNBC cell line, we hypothesized that PVT1 exon 9 may play a regulatory role in claudin expression in TNBC cells. To determine if PVT1 exon 9 plays a regulatory role in claudin expression, we examined the effect of SiRNA targeting of PVT1 exon 9 on messenger RNA (mRNA) and protein expression of claudins 1, 3, 4, and 7 in the claudin–low MDA MB 231 TNBC cell line. We observed that knockdown of PVT1 exon 9 in the claudin–low MDA MB 231 TNBC cell line does not significantly affect mRNA expression of claudins 1, 3, 4, and 7 when compared to control cells transfected with only control scramble non-targeting siRNAs (Figure 5). Similarly, we did not observe any significant change in protein expression of claudins 1, 3, and 7 when PVT1 exon 9 is knocked down. Interestingly, though, we observed re-expression of CLDN4 protein in the claudin–low MDA MB 231 TNBC cell line when PVT1 exon 9 expression is knocked down (Figure 6). These results suggest that PVT1 exon 9 is regulating claudin 4 protein expression in the claudin–low MDA MB 231 TNBC cell line.

### 2.5. PVT1 Exon 9 and Epithelial-Mesenchymal Transition (EMT) in Claudin–Low TNBC Cells

**Figure 7:**
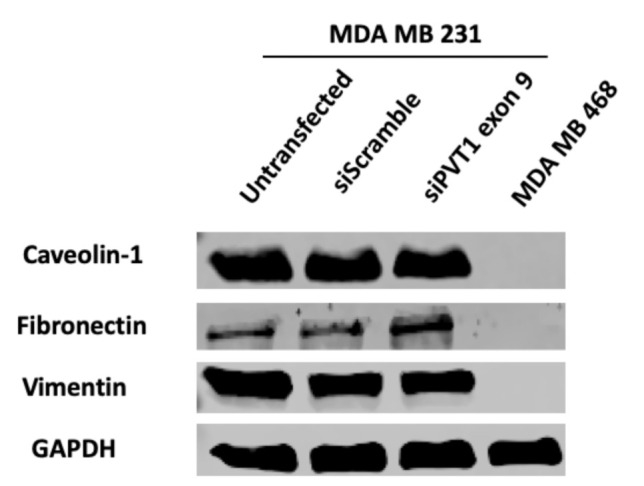
SiRNA targeting of PVT1 exon 9 does not affect EMT in MDA MB 231 CL TNBC cells. MDA MB 231 CL TNBC cells were transfected with PVT1 exon 9 specific siRNAs (siPVT1 exon 9) for 24 h. Western blotting was performed using specific antibodies against vimentin, caveolin and fibronectin. When compared to MDA MB 231 CL TNBC cells transfected with only control scramble non-targeting siRNA (siScramble). siRNA targeting of PVT1 exon 9 did not change the expression of EMT markers in MDA MB 231 CL TNBC cells, except for a slight increase in fibronectin.

Epithelial-mesenchymal transition (EMT) is a critical process that occurs in many types of cancers [52], and PVT1 has been shown to be involved in EMT induction [23]. To investigate if PVT1 exon 9 expression may be regulating EMT CL TNBC cells, western blotting was used to assess the protein expression of EMT markers (vimentin, E-cadherin, fibronectin and caveolin) in the MDA MB 231 CL TNBC cell line after siRNA knockdown of PVT1 exon 9. We observed no changes in EMT markers when PVT1 exon 9 is knocked down. However, our data indicates that EMT markers are more highly expressed in the MDA MB 231 CL TNBC cells in comparison to the MDA MB 468 CH TNBC cell line (Figure 7).

## 3. Discussion

Although much progress has been made in breast cancer management and treatment, patients with TNBC continue to have poor prognosis [4,58,59] with CL TNBC having the worst outcome among the subtypes of BC. Presently, efficient treatment remains unavailable for CL TNBC. Consequently, efforts towards understanding the molecular mechanisms which regulate CLDN expression in TNBC is imperative, as it could potentially uncover novel opportunities for the development of effective therapeutic strategies. Many studies have already demonstrated PVT1’s functional role in breast tumorigenesis [24]. However, there is a deficit of studies on the specific mechanisms by which PVT1 plays an important role in breast tumorigenicity. 

Differential expression of PVT1 alternatively spliced transcripts in breast cancer have not been previously investigated. The purpose of this study was to investigate the relationship between breast tumorigenesis and PVT1. In this study, we demonstrated that PVT1 may play an important regulatory role in TNBC. Particularly, our data indicates that PVT1 transcripts containing exon 9 may regulate claudin expression in claudin–low MDA MB 231 TNBC cells. Our group previously reported that PVT1 exon 9 was differentially expressed in prostate cancer. More specifically, PVT1 exon 9 was overexpressed in prostate cancer tissue [18]. Furthermore, PVT1 exon 9 expression was reported to be significantly higher in prostate cancer cell lines with an aggressive phenotype [17]. The implications of this study suggest that alternatively spliced transcripts of PVT1, including transcripts containing PVT1 exon 9, may be associated with increased risk of cancer. In a *previous* study, overexpression of PVT1 exon 9 induced malignant transformation, increased cell proliferation, and migration in prostate epithelial cells [19]. These studies established an oncogenic role for PVT1 exon 9 in prostate cancer. However, to our knowledge, the significance of PVT1 exon 9 in breast cancer was not previously investigated. 

Our data demonstrated that PVT1 exon 9 was significantly overexpressed in CL MDA MB 231 TNBC cells, and significantly under-expressed in CH MDA MB 468 TNBC cells, when compared to T47D cells. Based on these results, we used CL MDA MB 231 as a model for PVT1 exon 9 overexpression, and CH MDA MB 468 as a model for PVT1 exon 9 under-expression.

Cancer cell migration is a characteristic of metastasis and is associated with poor prognosis in cancer patients [24]. We observed that MDA MB 231 cells transfected with siPVT1 exon 9 were less migratory when compared to cells that were transfected using a scramble control. These results suggest that overexpression of PVT1 exon 9 increases the migratory capacity of the CL MDA MB 231 TNBC cells, and that loss of PVT1 exon 9 expression may have a protective role by making these cells less migratory. A more in-depth study in order to elucidate the underlying mechanisms by which this regulatory process occurs is necessary.

Differential expression of CLDNs are tissue and cell specific. Consequently, their functions are based on their localization and expression pattern. CLDNs 1, 3, 4 and 7 are among the most frequently dysregulated of the CLDN family members [30,60]. Based on other studies, we know that lncRNAs act as critical regulators of gene expression [61]. This is in keeping with our observation that PVT1 exon 9 is overexpressed in CL MDA MB 231 TNBC cells and under expressed in CH MDA MB 468 TNBC cells. We hypothesized that PVT1 may be regulating claudin expression either at a post-transcriptional level, a post-translational level, or indirectly. One way lncRNAs can serve as post transcriptional gene regulators is by forming ribonucleoprotein complexes via interacting directly with various RNA binding proteins (RBPs) to affect mRNA stability [62]. LncRNA PTOV1-AS1 interacts directly with hnRNPK in order to modulate HMOX1 expression [63]. At a post-translational level, lncRNAs can regulate protein stability by slowing down their degradation. LncRNA HOTAIR, for example, inhibited the interaction between AR and E3 ubiquitin ligase MDM2 after binding to AR [64]. 

Our confirmation of CLDNs 1, 3, 4 and 7 expression in MDA MB 231 cells is in keeping with previous reports. Knockdown of PVT1 exon 9 showed no significant changes in the expression of CLDN mRNA transcripts suggesting that in MDA MB 231 cells PVT1 exon 9 may not be regulating CLDN expression at a transcriptional level. PVT1 may be interacting with CLDN proteins directly, or indirectly, to regulate their expression.

LncRNAs are known to interact with proteins to regulate their stability, or ubiquitination [65]. Our data demonstrate that PVT1 regulates CLDN expression at a post-translational level. Knock down of PVT1 exon 9 did not affect protein expression for CLDNs 1, 3 and 7, however, we did observe re-expression of CLDN4 in MDA MB 231 cells. Since knockdown of PVT1 expression led to an increase in CLDN4 protein expression, it is plausible that PVT1 regulates CLDN4 protein expression via ubiquitination. Protein ubiquitination is one of the most common post-translational modification used to regulate various protein substrates in numerous cellular pathways. LncRNA MEG3, a tumor suppressor reported to play important roles in various malignancies, has been demonstrated to regulate LATS2 by promoting the ubiquitination of EZH2 in gallbladder cancer [66], while lncRNA HOTAIR acts as an inducer of proteolysis by facilitating the ubiquitination of Ataxin-1 and Snurportin-1. Over expression of HOTAIR promotes their rapid degradation [67]. Similarly, PVT1 overexpression may downregulate CLDN4 expression by regulating its ubiquitination. One explanation of how PVT1 could be doing this is by binding directly to CLDN4 thus facilitating its downregulation. However, an RNA immunoprecipitation (RIP) assay in which CLDN4 was used to pull down PVT1 exon 9 transcripts suggests otherwise, as there was no enrichment of PVT1 exon 9 transcript in the IP when compared to the control (Figure S6). Consequently, PVT1 may be regulating CLDN4 protein expression via an as yet undiscovered mechanism such as modulation of a downstream target, crosstalk between ubiquitination mediators, or by utilizing a different molecular mechanism altogether. Additional studies are necessary to further investigate this. Overexpression of CLDN4 is reported to have unfavorable clinical outcomes [57]. Contrastingly, the implications of our results suggest that re-expression of CLDN4 in MDA MB 231 CL TNBC cells is associated with a reduction in migration. This is in keeping with Lin et. al, who reported that loss of CLDN4 promotes EMT, while re-expression, or increased expression, of CLDN4 reduces migration [68]. Moreover, silencing PVT1 exon 9 had no significant effect on cell proliferation, suggesting that PVT1 may not have a regulatory role in cell proliferation (Figure S3).

The epithelial-to-mesenchymal transition (EMT) describes a biological process in which epithelial cells undergo a gradual change becoming more “mesenchymal-like”, motile and invasive [69]. Concomitant downregulation of epithelial markers, and upregulation of mesenchymal markers, is characteristic of EMT [70]. Aberrant expression of both CLDNs, and PVT1, induce EMT in many malignancies [71,72]. We assessed the expression of the following EMT markers: E-cadherin, vimentin and fibronectin. Furthermore, we assessed the expression of caveolin-1, an integral membrane protein that participates in several cellular processes including EMT [73]. We observed no changes in the expression of EMT markers when PVT1 exon 9 is knocked down, except for a slight increase in the expression of fibronectin. Fibronectin is a component of the extra cellular matrix whose upregulation is typically associated with increased migration in cancer cells [74,75]. Thus, the slight increase of fibronectin in our study may not be associated with the decrease in migration, but could potentially have implications for other biological processes involving PVT1. All in all, our data suggest that EMT markers are more highly expressed in MDA MB 231 cells in comparison to MDA MB 468 cells. Though this supports that MDA MB 231 CL TNBC cells are more “mesenchymal-like”, PVT1 exon 9 does not appear to have a role in this process. Overall, this result suggests that PVT1 may not regulate EMT in TNBC.

Keratin 14 (KRT14), a member of the keratin type I family is overexpressed in breast cancer [76,77,78]. Cells expressing KRT14 are more migratory [79] and invasive [80,81]. Interestingly, there is evidence which demonstrates that KRT interacts with CLDNs in order to maintain tight junction functionality [82]. Moreover, it was reported that modulation of KRTs affects CLDN expression, cell motility, and invasion in hepatocellular carcinoma [83]. Therefore, in future studies it may be worth investigating PVT1 regulation of CLDN4 in MDA MB 231 CL TNBC cells as part of a potentially novel pathway involving KRT14.

## 4. Materials and Methods

### 4.1. Cell Culture

A panel of five breast cancer cell lines were used to assess the expression of PVT1 exon 9. MDA MB 231, MDA MB 468, T47D and MCF-7 were maintained in Dulbecco’s Modified Eagle’s Medium (DMEM), supplemented with 10% FBS and 0.5% penicillin/streptomycin. Trypsinization of cells occurred using 0.05% trypsin when cells were 70–80% confluent. BT474 was maintained in F12/DMEM (GIBCO, Waltham, MA, USA), supplemented with 10% FBS and 1% penicillin/streptomycin. Trypsinization of cells occurred using 0.25% trypsin when cells were 70–80% confluent. All cell lines were cultured in a 5% CO_2_, 37 °C atmosphere.

### 4.2. Transfection of siRNAs

MDA MB 231 cells were grown in 6-well plates until they have reached 90–100% confluency. According to the manufacturer’s instructions, cells were transfected with 10 nM of PVT1 exon 9 siRNA (siPVT1 exon 9) (Forward: 5′ ACCUAUGAGCUUUGAAUAA 3′; Reverse: 5′ UUAUUCAAAGCUCAUAGGU 3′) (Sigma, St. Louis, MO, USA), or a non-targeting scramble control (siScramble) (Forward: 5′ CUCACUACCGUCGACCCCA 3′; Reverse: 5′ UGGGGUCGACGGUAGUGAG 3′) (Sigma) using Lipofectamine RNAiMAX (Thermo Fisher Scientific Inc.; Wilmington, DE, USA). siRNAs and Lipofectamine were diluted in Opti-MEM (Thermo Fisher Scientific Inc.). Following transfection, cells were incubated for 24 h in a 5% CO_2_, 37 °C atmosphere before being harvested, or migration assay.

### 4.3. RNA Extraction and RT-qPCR

At 70–75% confluency, total RNA was extracted from cells in a 60 × 15 mm tissue culture dish, using the RNeasy Mini Kit (Qiagen, Hilden, Germany, cat#: 74104) according to the manufacturers’ instructions. RNA concentration was measured using the spectrophotometer NanoDropTM 2000 (Thermo Fisher Scientific, Inc.). cDNA was synthesized from 1 μg of RNA using QuantiTect reverse transcription kit (Qiagen, cat# 205311). Amplification reactions were performed in 25 μL reaction volume using SYBR Green PCR master mix (Life Technologies, Grand Island, NY, USA cat# 4309155), cDNA template and 0.4 μM final concentration for primers. Primers used in this study were composed of the following oligonucleotide sequences listed in Table S1. Using the Quantifect Studio System (Applied Biosystems), relative expression of messenger RNA (mRNA) for each sample was assessed in quadruplicates in at least 3 independent experiments, and quantified via the comparative cycle threshold (∆∆ Ct) method and normalized to GAPDH mRNA expression.

### 4.4. Protein Extraction and Immunoblotting

Whole cell extracts were obtained using RIPA lysis buffer (VWR Life Science, Radnor, PA, USA, cat# N653-100ML) supplemented with 1× protease inhibitor and 100 mM of phenyl methylsulfonyl fluoride (PMSF) (Amresco, Solon, OH, USA, cat# M145-5G). Protein concentration was quantified via the Bradford Assay using the Bio-Rad Protein Assay Dye Reagent Concentrate (BioRad, Hercules, CA, USA, cat# 500-0205). For western blot analysis, 50 μg of protein were resolved by 15% SDS-PAGE gels and subsequently transferred onto nitrocellulose membranes. Membranes were blocked in 5% BSA or 5% milk in TBS-T for 1 h at room temperature, incubated overnight at 4 °C in primary antibodies. Next, membranes were washed in 1× TBS-T, incubated with secondary antibodies for 2 h, washes with 1× TBS-T and imaged using the Odyssey CLx imager with infrared fluorescence (LI-COR, Lincoln, NE, USA). The primary antibodies used were Claudin 1 (13050-1-AP), Claudin 4 (16195-1-AP) and Claudin 7 (10118-1-AP) (Proteintech, Rosemont, IL, USA), Claudin 3 (341700; Invitrogen, Waltham, MA, USA), GAPDH (5174S; 1:1000; Cell Signaling, Danvers, MA, USA), α-tubulin (sc-32293; 1:1000; Santa Cruz Biotechnology, Dallas, TX, USA). The secondary antibodies used were anti-mouse (925-32210; 1:15,000; LI-COR) and anti-rabbit (925-32211; 1:15,000; LI-COR). Original blots can be found at Figures S1 and S2.

### 4.5. Migration Assays

1 × 10^5^ MDA MB 231 cells were grown in a 6-well tissue culture plate until 90–100% confluency. Cells were transfected with siRNA, or siScramble, and incubated at 5% CO_2_, 37 °C for 24 h. Wounds were made on the cell monolayer using a sterile 200 μL pipet tip and then washed with 1× PBS and incubated with media containing respective siRNAs. Images of scratched areas were taken at 10× magnification using an AE30 inverted microscope (Motic, Richmond, BC, Canada).

### 4.6. Cell Viability Assays

10^4^ cells were seeded into 96 well plates. At 70% confluency, the cells were transfected with PVT1 exon 9. After 24 h, MTT assays were performed and absorbance measured at 490 nm with a microplate reader (i3 multimode microplate reader, Spectramax, San Jose, CA, USA).

### 4.7. Crosslinking and RNA Immunoprecipitation (RIP)

RIP experiments were performed using MDA MB 231 cells. Cells were plated in 10 cm tissue culture dishes until 90–100% confluent. Cells were trypsinized and resuspended in culture medium. RNA was crosslinked to proteins by adding formaldehyde drop-wisely to suspended cells at a final concentration of 0.75%. Cells were placed on a shaker at a low speed for 10 min at room temperature. A final concentration of 125 mM of glycine was added to the media and incubated at room temperature on a shaker for 5 min), and then pelleted. Cells were resuspended in 2 mL freshly prepared nuclear isolation buffer (1.28 M sucrose, 40 mM Tris-HCl [pH 7.5], 20 mM MgCl2, 4% Triton X-100), 2 mL 1× PBS and 6 mL nuclease free water, and kept on ice for 20 min with frequent mixing. Cells were pelleted at 2500 *g* for 15 min and resuspended in 1 mL freshly prepared RIP buffer (1861603, Thermo Fisher Scientific Inc.) supplemented with 1× protease inhibitor, 100 U/mL RNase inhibitor (AM2696, Invitrogen, Waltham, MA, USA) and 0.5 mM DTT, for 10 min on ice. Lysate was then sonicated for 3 min (97 amplitude, 30 s on, 30 s off) and centrifuged at 13,000 rpm at 4 °C for 10 min. 40 uL of Dynabeads protein G (10007D, Invitrogen) was washed once with wash buffer and incubated with 10 ug of CLDN4 antibody (16195-1-AP, Proteintech) for 1 h at room temperature. Beads were washed again with wash buffer and incubated with cell lysate for 2 h at 4 °C. Following incubation, beads were washed 3 times with wash buffer and once with 1× PBS. RNA was extracted using Trizol (10296010, Ambion, Austin, TX, USA) as per manufacturers’ recommendation. cDNA was synthesized from 150 ng of RNA using QuantiTect reverse transcription kit (Qiagen, cat# 205311). Amplification reactions were performed in 25 μL reaction volume using SYBR Green PCR master mix (Life Technologies, Grand Island, NY, USA cat# 4309155), cDNA template and 0.4 μM final concentration for primers. Using the Quantifect Studio System (Applied Biosystems, Foster City, CA, USA), relative expression of messenger RNA (mRNA) for each sample was assessed in quadruplicates in at least 2 independent experiments, and quantified via the comparative cycle threshold (∆∆ Ct) method.

### 4.8. Statistical Analysis

Data from at least three independent experiments were presented as mean ± standard error of the mean (S.E.M). Statistical significance was assessed using a two-tailed Student’s *t* test. *p* values less than 0.05 were deemed significant.

## 5. Conclusions

In conclusion, we demonstrated the potential regulatory role of PVT1 in CL MDA MB 231 TNBC cells by targeting PVT1 exon 9. Knock down of PVT1 exon 9 resulted in the re-expression of CLDN4 protein. Additionally, we demonstrated that overexpression of PVT1 exon 9 is associated with increased migration of MDA MB 231 CL TNBC cells. Our data suggest that PVT1 may have a regulatory role in CL MDA MB 231 TNBC cells by acting as a modulator of CLDN4 protein expression. These data may have implications for prognostication and treatment strategies in CL TNBC.

## Data Availability

All data generated from this study are contained in the manuscript and supplementary materials.

## Supplementary Materials

The following are available online at https://www.mdpi.com/2072-6694/13/5/1046/s1, Figure S1: The following are the corresponding blots to Figure 6. Figure S2: The following are the corresponding blots to Figure 7. Figure S3: PVT1 exon 9 does not regulate cell viability. Figure S4: PVT1 exon 9 regulates migration of MDA MB 231 CL TNBC cells. Figure S5: PVT1 exon 9 is overexpressed in MDA MB 231 cells. Figure S6: PVT1 exon 9 does not bind directly to CLDN4. Table S1: List of primers sequences.
