# Supplementary material for: Targeting PVT1 Exon 9 Re-Expresses Claudin 4 Protein and Inhibits Migration by Claudin—Low Triple Negative Breast Cancer Cells
Source: Cancers (Basel). 2021 Mar 2;13(5):1046. doi: 10.3390/cancers13051046 (PMC7958609; doi:10.3390/cancers13051046)
Supplement: Supplementary file 1 [file cancers-13-01046-s001.pdf]

Supplementary Materials:

Targeting PVT1 Exon 9 Re-Expresses Claudin 4 Protein and Inhibits Migration by Claudin–Low Triple Negative Breast Cancer Cells

Fayola Levine and Olorunseun O. Ogunwobi

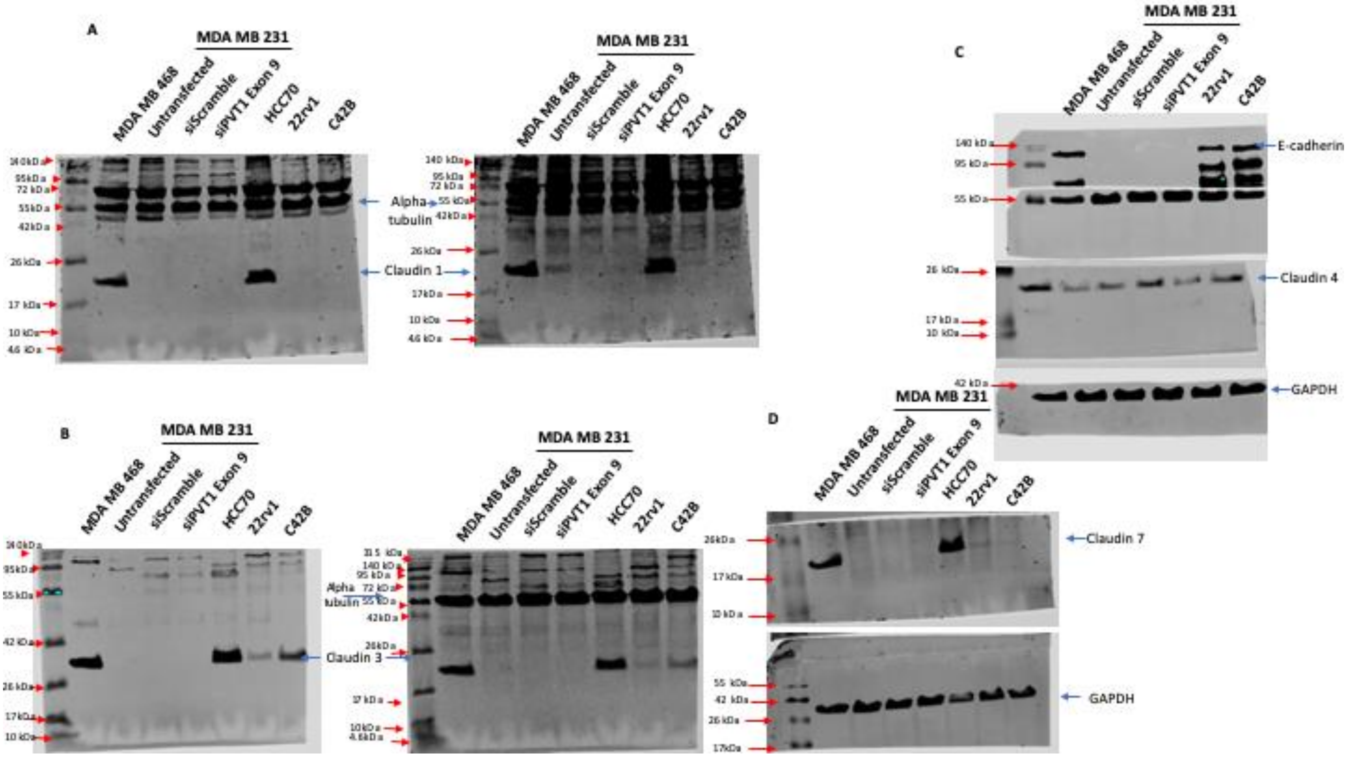

Figure S1. The following are the corresponding blots to figure 6. SiRNA targeting of PVT1 exon 9 induces claudin 4 protein re-expression in MDA MB 231 CL TNBC cells. MDA MB 231 CL TNBC cells were transfected with PVT1 exon 9 specific siRNAs (siPVT1 exon 9) for 24 hours. Western blotting was performed using specific antibodies against claudin 1, claudin 3, claudin 4, claudin 7 and E-Cadherin. SiRNA targeting of PVT1 exon 9 induced claudin 4 protein re-expression in MDA MB 231 CL TNBC cells in comparison to MDA MB 231 CL TNBC cells transfected with only control scramble non-targeting siRNA (siScramble).

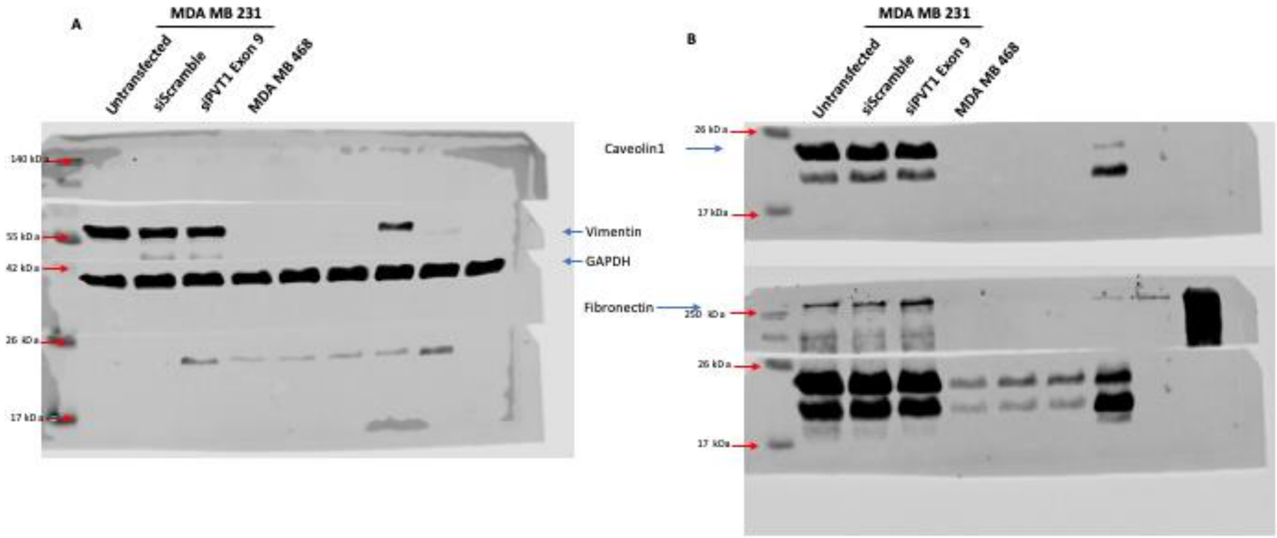

Figure S2. The following are the corresponding blots to figure 7. SiRNA targeting of PVT1 exon 9 does not affect EMT in MDA MB 231 CL TNBC cells. MDA MB 231 CL TNBC cells were transfected with PVT1 exon 9 specific siRNAs (siPVT1 exon 9) for 24 hours. Western blotting was performed using specific antibodies against vimentin, caveolin and fibronectin. When compared to MDA MB 231 CL TNBC cells transfected with only control scramble non-targeting siRNA (siScramble), siRNA targeting of PVT1 exon 9 did not change the expression of EMT markers in MDA MB 231 CL TNBC cells.

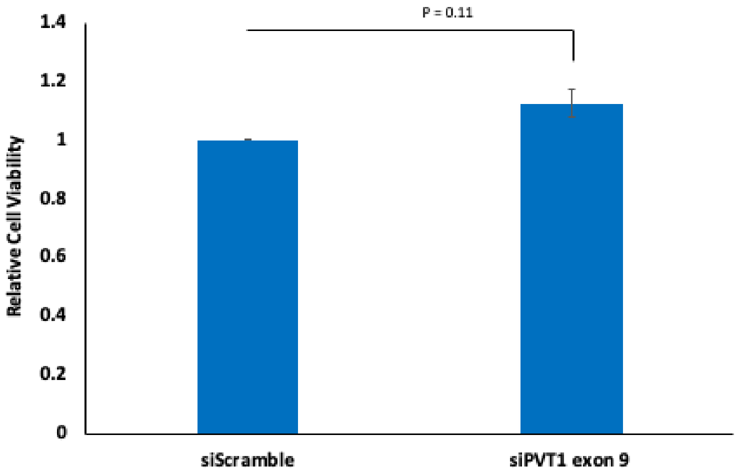

Figure S3. PVT1 exon 9 does not regulate cell viability. MDA MB 231 cells were transfected with siRNA targeting PVT1 exon 9 for 24 hours, followed by an MTT assay. When compared to a control scramble non-targeting siRNA (siScramble), cells transfected with siPVT1 exon 9 had a slight increase in cell viability. However, the change was not significant. Statistical differences are presented as mean ± s.d; *t*-tests, $p = 0.11$, and is not significant. Quantification of differences in cell viability is based on at least 3 independent experiments.

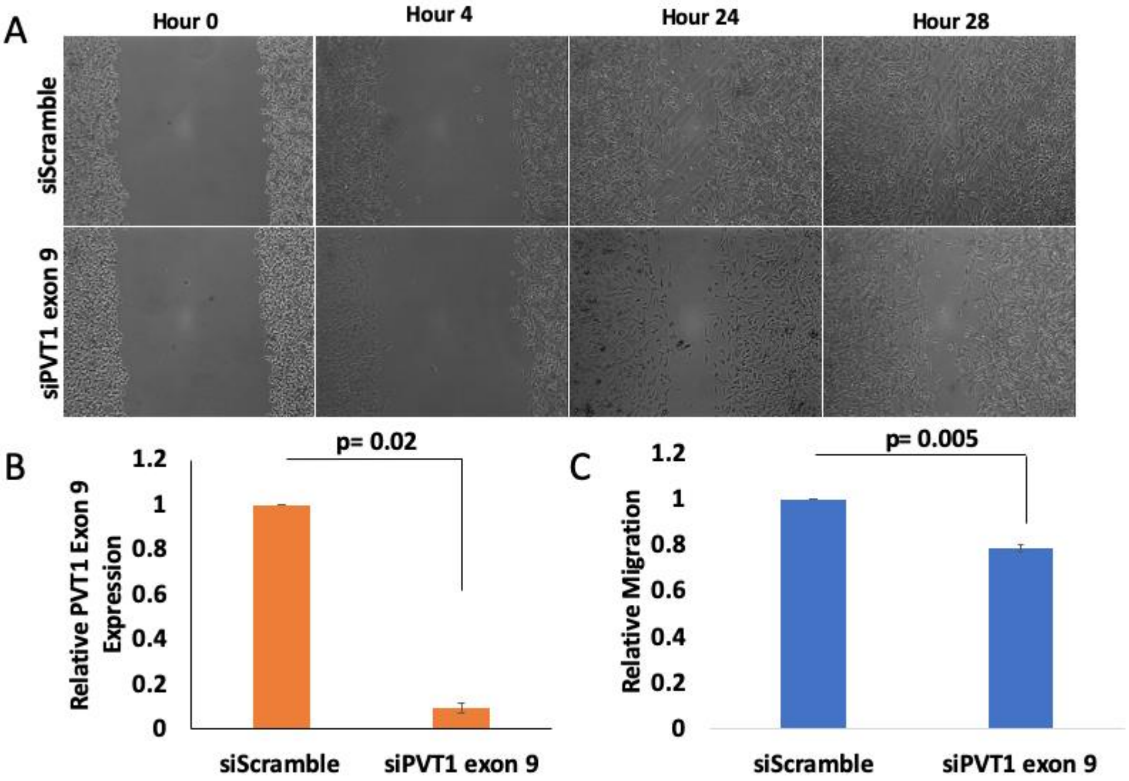

Figure S4. PVT1 exon 9 regulates migration of MDA MB 231 CL TNBC cells. (A) Wound healing migration assays were performed with the MDA MB 231 CL TNBC cell line. MDA MB 231 cells were transfected once confluent. After 24 hours, wounds were made and pictures were taken at 0 hours, 4 hours, 24 hours, and 28 hours. magnification (B) Knockdown of PVT1 exon 9 expression in the MDA MB 231 CL TNBC cell line at hour 0. Transfection of SiRNAs that specifically target PVT1 exon 9 was performed. Relative expression of PVT1 exon 9 in MDA MB 231 cells was assessed, based on data from 2 independent experiments. (C) Quantification of differences in migration, after 28 hours, based on data from 3 independent experiments.

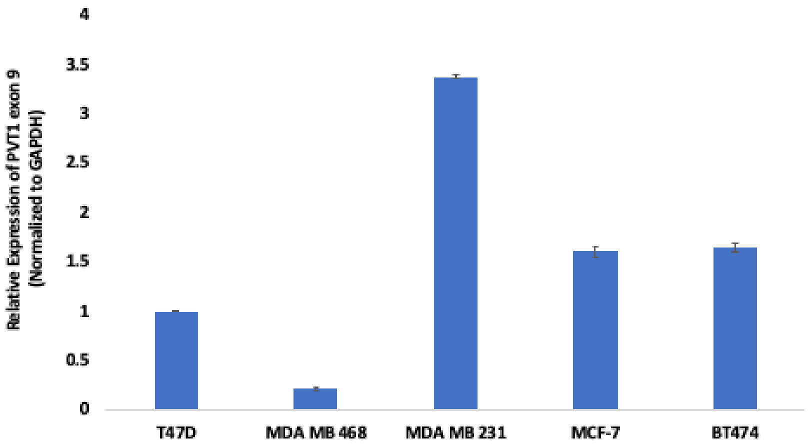

Figure S5. PVT1 exon 9 is overexpressed in MDA MB 231 cells. Comparison of PVT1 exon 9 expression in the T47D (ER +, PR+, HER2+) BC cell line, MDA MB 468 CH TNBC cell line, MCF-7 CH TNBC cell line, BT474 CH TNBC cell line and MDA MB 231 cell CL TNBC cell line. PVT1 exon 9 expression was assessed using RT-qPCR in the T47D estrogen receptor positive BC cell line, MDA MB 231 CL TNBC cell line and in the MDA MB 468 CH TNBC cell line. Expression was normalized against GAPDH. Data presented as mean ± s.d. Results are from experiments performed in quadruplicates 6 separate times.

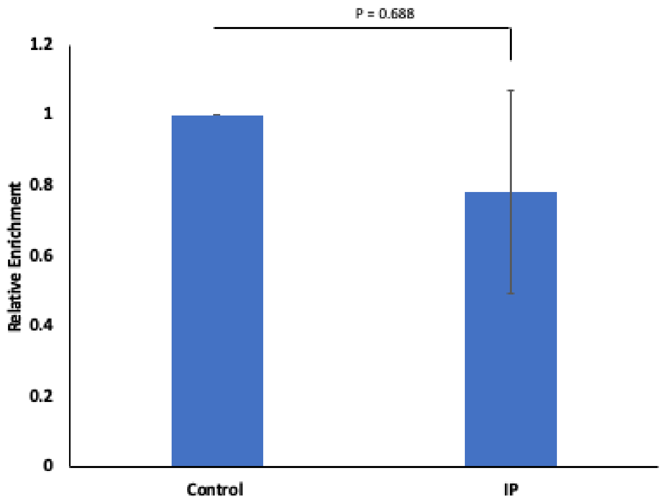

Figure S6. PVT1 exon 9 does not bind directly to CLDN4. RNA immunoprecipitation (RIP) of PVT1 exon 9. Protein G Dyna beads were incubated with 10 ug of CLDN4 antibodies for 1 hour at room temperature, followed by 2 hours incubation with cell lysate at 4 °C (IP). RNA eluates were reverse - transcribed and assessed via RT q-PCR. All enrichments are normalized to the input sample and control. Each RIP experiment was performed on two independent biological replicates. Data are presented as mean ± s.d.; *t*-tests: $p = 0.688$ and is not significant.

Table 1. List of primer sequences.

Primer Name	Primer Sequence 5'-3'
PVT1 exon 9-F	CATGACTCCACCTGGACCTT
PVT1 exon 9-R	GTGGGCGATGAAGTTCGTA
CLDN1-F	CTGCTGCTTCTCTCTGCCTT
CLDN1-R	GCAGGTTTTGGATAGGGCCT
CLDN3-F	GGACTTCTACAACCCCGTGG
CLDN3-R	TGGTGGCCGTGTACTTCTTC
CLDN4-F	TGGGAGGGCCTATGGATGAA
CLDN4-R	GCTTTCATCCTCCAGGCAGT
CLDN7-F	GTCTTGCCACCTTGGTAGCT
CLDN7-R	CCCTGCCCAGCCAATAAAGA
GAPDH-F	GAGTCAACGGATTTGGTCGT
GAPDH-R	TTGATTTTGGAGGGATCTCG
